# The elastic solid solution model for minerals at high pressures and temperatures

**DOI:** 10.1007/s00410-017-1436-z

**Published:** 2018-01-23

**Authors:** R. Myhill

**Affiliations:** School of Earth Sciences, University of Bristol, Bristol, UK

**Keywords:** High pressure, Excess properties, Solution model, Solid, Elasticity

## Abstract

Non-ideality in mineral solid solutions affects their elastic and thermodynamic properties, their thermobaric stability, and the equilibrium phase relations in multiphase assemblages. At a given composition and state of order, non-ideality in minerals is typically modelled via excesses in Gibbs free energy which are either constant or linear with respect to pressure and temperature. This approach has been extremely successful when modelling near-ideal solutions. However, when the lattice parameters of the solution endmembers differ significantly, extrapolations of thermodynamic properties to high pressures using these models may result in significant errors. In this paper, I investigate the effect of parameterising solution models in terms of the Helmholtz free energy, treating volume (or lattice parameters) rather than pressure as an independent variable. This approach has been previously applied to models of order–disorder, but the implications for the thermodynamics and elasticity of solid solutions have not been fully explored. Solid solution models based on the Helmholtz free energy are intuitive at a microscopic level, as they automatically include the energetic contribution from elastic deformation of the endmember lattices. A chemical contribution must also be included in such models, which arises from atomic exchange within the solution. Derivations are provided for the thermodynamic properties of *n*-endmember solutions. Examples of the use of the elastic model are presented for the alkali halides, pyroxene, garnet, and bridgmanite solid solutions. Elastic theory provides insights into the microscopic origins of non-ideality in a range of solutions, and can make accurate predictions of excess enthalpies, entropies, and volumes as a function of volume and temperature. In solutions where experimental data are sparse or contradictory, the Helmholtz free energy approach can be used to assess the magnitude of excess properties and their variation as a function of pressure and temperature. The formulation is expected to be useful for geochemical and geophysical studies of the Earth and other planetary bodies.

## Introduction

Thermodynamic and thermoelastic models of minerals and melts underpin our knowledge of the structure and dynamics of the Earth, its evolution through time, and the causes of seismic velocity variations in the deep interior. The physical properties of mineral endmembers are usually well constrained by a significant body of experimental and theoretical work to high pressure and temperature. However, most geologically interesting phases span a compositional range, which can be described as a solid solution of distinct endmembers. The variation of physical properties across these solid solutions is often less well constrained than the properties of the bounding endmembers, and must be approximated (Davies and Navrotsky 1983; Powell et al. 2014).

If the endmembers of a solid solution are structurally, volumetrically, and chemically similar, mixing can be approximated as ideal. In an ideal solution model, the non-configurational contributions to the total potentials (internal energy, enthalpy, Gibbs free energy, and Helmholtz free energy) are equal to the sum of endmember potentials multiplied by the molar fractions of each endmember. Unfortunately, for many geologically interesting minerals, mixing is non-ideal, and excess enthalpies $\mathcal {H}_{\text {excess}}$, volumes $V_{\text {excess}}$ and/or entropies $S_{\text {excess}}$ are often observed (e.g., Kerrick and Darken 1975). Constraining these excess properties has proven difficult, both because impurities, ordering, and poor crystallinity can influence excess properties, and because of the challenging nature of the experiments required to measure those excesses. Indeed, the differences between estimates of excess properties reported by different research groups are often several times the reported measurement errors (c.f. Berman 1990). In addition, most measurements of excess properties are confined to room pressure and low temperature, far from the conditions of interest. Relatively little attention has been paid to the pressure and temperature dependence of these excess properties.

In the Earth Sciences, thermodynamic models of solid solutions have typically been formulated as explicit functions of the Gibbs free energy. This is true both of disordered models appropriate at high temperatures (Stixrude and Lithgow-Bertelloni 2011; Holland et al. 2013) and of models including order–disorder (e.g., Carpenter 1988; Ghiorso 1990; Putnis 1992; Salje 1993; Holland and Powell 1996; Ghiorso and Evans 2002; Holland and Powell 2006). In these models, parameter values describing interaction energies are typically either constant, or linear functions of pressure and temperature. This is equivalent to assuming that excess non-configurational volumes and entropies are zero or pressure-temperature independent. Excess volumes and entropies must approach zero at high pressure and low temperature, respectively, and it is unclear to what extent constant non-zero excesses impact thermodynamic and elastic properties at geologically interesting conditions. Furthermore, several published studies suggest that excess entropies and volumes are dependent on pressure and temperature (e.g., Andrault et al. 2007; Benisek and Dachs 2012; Du et al. 2015). An understanding of the robustness and potential origins of these observations would be particularly useful for seismic studies which use thermodynamic models at high pressures and temperatures (Sanloup et al. 2000, 2004; Davies et al. 2012; Mosca et al. 2012; Deschamps et al. 2012; Gudkova et al. 2014).

Some studies of non-ideal solutions have moved beyond empirical fitting by specifically considering the energetic consequences of mixing dissimilar endmembers. The models developed are typically still parameterised in terms of the Gibbs free energy, but the parameterizations include a consideration of the elastic energy required to match the volumes of the endmember lattices at 1 bar (e.g., Ferreira et al. 1988; Ganguly et al. 1996; Urusov 2000). In this study, I show that this technique is equivalent to reformulating non-ideality as a function of the Helmholtz free energy, which has long been recognised as a natural potential to use for solid–solid reactions (e.g., Landau 1937; Dove 1997; Hobbs and Ord 2016).

I provide several examples which illustrate that the use of the Helmholtz free energy decreases the number of empirically fitted parameters required to fit experimental data, and, therefore, has greater predictive power than the traditional Gibbs free energy approaches. For pyrope-grossular garnet, the model is able to predict the magnitude and pressure–temperature dependence of the excess non-configurational entropy, which is extremely difficult to constrain experimentally. The implementation introduced in the following sections allows for the addition of further parameters where they are required to fit experimental data.

## Excess thermodynamic properties of solutions

### Non-ideality in Gibbs free energy

In an ideal solution composed of multiple endmembers with molar proportions $p_i$, molar volumes are linearly dependent on composition (*X*) at fixed pressure (*P*) and temperature (*T*). Any non-ideality in volume is incorporated via an excess term:

1 $$\begin{aligned} V(P, T, X) = \sum _ip_i V_i(P, T) + V_{\text {excess}}(P, T, X). \end{aligned}$$

To satisfy the thermodynamic identity $\left( \frac{\partial \mathcal {G}}{\partial P} \right) _{T}= V$, the non-configurational molar Gibbs free energy $\mathcal {G}$ of an ideal solution must also be linearly dependent on composition. The Gibbs free energy of a generalised non-ideal solution is, therefore:

2 $$\begin{aligned} \mathcal {G}(P, T, X) = \sum _ip_i \mathcal {G}_i(P, T) + \mathcal {G}_{\text {excess}}(P, T, X), \end{aligned}$$

where the excess energy term accounts for any non-ideality and configurational energy. The thermodynamic properties of a solid solution are found by appropriate differentiation of Eq. 2:

3 $$\begin{aligned} S(P, T, X)= -\left( \frac{\partial \mathcal {G}}{\partial T} \right) _{P} = \sum _ip_iS_i - \left( \frac{\partial \mathcal {G}_{\text {excess}}}{\partial T} \right) _{P} \end{aligned}$$

4 $$\begin{aligned} V(P, T, X)= \left( \frac{\partial \mathcal {G}}{\partial P} \right) _{T} = \sum _ip_iV_i + \left( \frac{\partial \mathcal {G}_{\text {excess}}}{\partial P} \right) _{T} \end{aligned}$$

5 $$\begin{aligned} K_{T}(P, T, X)= -V \left( \frac{\partial ^2 \mathcal {G}}{\partial {P}^2} \right) _{T}^{-1} = V \left( \sum _i\left( p_i \frac{V_{i}}{K_{Ti}} \right) - \left( \frac{\partial ^2 \mathcal {G}_{\text {excess}}}{\partial {P}^2} \right) _{T} \right) ^{-1} \end{aligned}$$

6 $$\begin{aligned} C_P(P, T, X)= -T \left( \frac{\partial ^2 \mathcal {G}}{\partial {T}^2} \right) _{P} = \sum _ip_iC_{pi} -T \left( \frac{\partial ^2 \mathcal {G}_{\text {excess}}}{\partial {T}^2} \right) _{P} \end{aligned}$$

7 $$\begin{aligned} \alpha (P, T, X)= \frac{1}{V} \left( \frac{\partial V}{\partial T} \right) _{P} = \frac{1}{V} \left( \sum _i\left( p_i\,\alpha _i\,V_i \right) + \frac{\partial ^2 \mathcal {G}_{\text {excess}}}{\partial {P} \partial {T}} \right) . \end{aligned}$$

Finally, the other thermodynamic properties can be obtained using the usual identities:

8 $$\begin{aligned} \mathcal {F}= \mathcal {G} - PV \end{aligned}$$

9 $$\begin{aligned} C_{V}= C_{P} - V\,T\,\alpha ^{2}\,K_{T} \end{aligned}$$

10 $$\begin{aligned} K_{S}= K_{T} \,\frac{C_{P}}{C_{V}} \end{aligned}$$

11 $$\begin{aligned} \gamma= \frac{\alpha \,K_{T}\,V}{C_{V}}. \end{aligned}$$

A wide variety of different models for $\mathcal {G}_{\text {excess}}$ have been proposed. The simplest of these models assume complete disorder in the solid solution, and take the form $\mathcal {G}_{\text {excess}} = \mathcal {E}_{\text {excess}} + P V_{\text {excess}} - T S_{\text {excess}}$, where the excess energy, volume, and entropy terms are constants. The entropy term accounts for both the configurational entropy and any non-configurational contribution. The compositional dependence of the excess free energy can be parameterised in a number of different ways, via (sub)regular (Helffrich and Wood 1989), (a)symmetric (Holland and Powell 2003; Diener et al. 2007), and Redlich–Kister models (Prausnitz et al. 1998), amongst others. If certain parameters are not well constrained by the existing measurements, they are commonly taken to be equal to zero or approximated using the excesses observed for the same element exchange in other phases (e.g., Powell et al. 2014).

In many natural solid solutions, order–disorder processes are important at the conditions of interest, and the excess energy and entropy terms can no longer be treated as constant (Sack 1980; Sack and Ghiorso 1989; Ghiorso 1990). Common macroscopic treatments of order–disorder include the highly successful “Landau” (Carpenter 1988; Putnis 1992; Salje 1993) and generalised “Bragg–Williams”-type models (Nell and Wood 1989; Ghiorso 1990; Putnis 1992; Holland and Powell 1996). In the Landau model, it is assumed that the “Landau” free energy ($\mathcal {L}$) of a transition is a polynomial expansion of the order parameter (*Q*). In the geological sciences, $\mathcal {L}$ is usually equated with the Gibbs free energy, but it is more correctly related to the Helmholtz free energy (Dove 1997). Typically, this equation takes the form:

12 $$\begin{aligned} \mathcal {L} = -HQ + \frac{1}{2}a(T - T_\mathrm{c})Q^2 + \frac{1}{4}bQ^4 + \frac{1}{6}cQ^6 + \cdots , \end{aligned}$$

where the field term *H* allows for non-convergent ordering (Carpenter et al. 1994) and $T_\mathrm{c}$ is the critical temperature, which may be pressure-/volume-dependent. In contrast, Bragg–Williams models explicitly add ordered members to the solid solution, and minimise the Gibbs free energy by varying the amount of the ordered and disordered endmembers subject to the bulk composition constraints. Both the Landau and Bragg–Williams models are capable of accurately reproducing experimentally derived variations in the state of order and configurational entropy (Holland and Powell 1996).

One important contributor to non-ideality that is not included in the above models is the elastic energy required to deform the endmembers to form the solid solution. The very act of mixing two dissimilar endmembers involves expansion, contraction, and more generally distortion of the endmember lattices. In the following sections, the non-ideal model is reformulated to investigate the contribution of this effect to the free energy, and its pressure and temperature dependence.

### The elastic model

Let us follow the logic of Ferreira et al. (1988) in approximating the elastic energy associated with creating a solid solution. It is assumed that the proportion of each endmember is sufficiently large that the exchanging atoms have overlapping strain fields, and, therefore, cannot be treated as impurities (Carpenter et al. 1999). In very dilute solid solutions, elastic strain energies are local and should be treated as such (Wood and Blundy 1997; Carpenter et al. 1999).

Consider two isotropic endmember minerals *A* and *B*, held at 0 K, where endmember *A* has a smaller equilibrium volume than endmember *B*. Forming the solution $A_xB_{1-x}$, therefore, requires an expansion of lattice *A* and contraction of lattice *B*. Each of these operations is associated with a change in elastic energy. In the absence of chemical interaction between the two lattices (i.e., if we do not break or form any new bonds), a solution of *A* and *B* at a given volume will have an elastic energy equal to the molar weighted sum of the endmember elastic energies:

13 $$\begin{aligned} \mathcal {U}_{\text {elastic}}(V, X) = \sum _ip_i \mathcal {U}_i (V). \end{aligned}$$

This static model can be extended to high temperature by assuming that the phonon density of states of the solution is equal to the molar weighted sum of the endmember densities of state, evaluated at volume *V*. The resulting entropy *S* of the solution is then equal to the molar weighted sum of the endmember entropies (Kieffer 1979). Using the relationship between the internal and Helmholtz free energies ($\mathcal {F} = \mathcal {U} - TS$), an analogue to Eq. 2 is derived as follows:

14 $$\begin{aligned} \mathcal {F}(V, T, X) = \sum _ip_i \mathcal {F}_i(V, T) + \mathcal {F}_{\text {excess}} (V, T, X). \end{aligned}$$

The properties of the solid solution at any fixed volume and temperature are found by partial differentiation of Eq. 14:

15 $$\begin{aligned} S(V, T, X)= -\left( \frac{\partial \mathcal {F}}{\partial T} \right) _{V} = \sum _ip_i S_i - \left( \frac{\partial \mathcal {F}_{\text {excess}}}{\partial T} \right) _{V} \end{aligned}$$

16 $$\begin{aligned} P(V, T, X)= -\left( \frac{\partial \mathcal {F}}{\partial V} \right) _{T} = \sum _ip_i P_i - \left( \frac{\partial \mathcal {F}_{\text {excess}}}{\partial V} \right) _{T} \end{aligned}$$

17 $$\begin{aligned} K_T(V, T, X)= V \left( \frac{\partial ^2 \mathcal {F}}{\partial {V}^2} \right) _{T} = \sum _i p_i K_{Ti} + V \left( \frac{\partial ^2 \mathcal {F}_{\text {excess}}}{\partial {V}^2} \right) _{T} \end{aligned}$$

18 $$\begin{aligned} C_V(V, T, X)= -T \left( \frac{\partial ^2 \mathcal {F}}{\partial {T}^2} \right) _{V} = \sum _i p_i C_{Vi} - T \left( \frac{\partial ^2 \mathcal {F}_{\text {excess}}}{\partial {T}^2} \right) _{V} \end{aligned}$$

19 $$\begin{aligned} \alpha (V, T, X)= - \frac{1}{K_T} \frac{\partial ^2 \mathcal {F}}{\partial {V} \partial {T}} = \frac{1}{K_T} \left( \sum _i p_i \alpha _i K_{Ti} - \frac{\partial ^2 \mathcal {F}_{\text {excess}}}{\partial {V} \partial {T}} \right) . \end{aligned}$$

The other thermodynamic properties ($C_P$, $K_S$, $\gamma$) can be found using Eqs. 9–11. A generalisation of the isotropic model to arbitrary stress fields and anisotropic endmembers is given in Appendix A, which yields an expression for the shear modulus, again as a function of volume, temperature and composition:

20 $$\begin{aligned} G(V, T, X) = \sum _i p_i G_{i}. \end{aligned}$$

If $\mathcal {F}_{\text {excess}}$ is a constant, an expression can also be derived for the variation in $K'$ across the solution:

21 $$\begin{aligned} K'(V, T, X) = \frac{\sum _i p_i K_i K'_i}{\sum _i p_i K_i}. \end{aligned}$$

To calculate the non-ideality of an elastic solution with endmember proportions $p_i$ at a given pressure and temperature, one must first solve Eq. 16 to find the equilibrium volume (although an approximate solution can be found using the expressions in Appendix B). Even when endmember volumes differ by as much as 10% (as is the case for highly non-ideal binary solutions such as pyrope-grossular), the thermodynamic deviations from ideality are almost quadratic with composition, justifying use of the regular solution model (e.g., Helffrich and Wood 1989). Interaction parameters for these models can be constrained using the excesses calculated at the midpoint of each binary (and ternary/higher order) system. For example:

22 $$\begin{aligned} W_{\mathcal {E}}^{AB}(P, T) \sim 4\mathcal {E}_{\text {excess}}^{A_{50}B_{50}}(P, T). \end{aligned}$$

In the above derivation, I have ignored the chemical mixing which must take place during solid solution formation. For example, in the case of the simple A–B binary, some of the A–A and B–B bonds in the endmember lattices will be replaced with A–B bonds. Such bonds are usually intermediate in length, and as such this “chemical” contribution to mixing typically reduces the non-ideality of the system (Ferreira et al. 1988). The magnitude and compositional dependence of $\mathcal {F}_{\text {excess}}(V, T)$ are discussed in “The chemical contribution to the excess Helmholtz energy”.

### The chemical contribution to the excess Helmholtz energy

#### Short-range clustering

The excess Helmholtz energy $\mathcal {F}_{\text {excess}}(V, T, X)$ (Eq. 14) is attributable to changes in bonding and structure resulting from mixing of the independent endmembers at constant volume and temperature (Sect. “The elastic model”). There are two components to this mixing. The exchange of atoms on particular sites in the lattice creates bonds which are distinct from those in the endmember lattices. In complex structures, there is also a potential energy contribution from distortion and tilting of structural groups within the lattice.

As a first approximation, let us assume that the chemical effects of mixing a set of endmember lattices (*A*, *B* ...) are dominated by short-range bonding within clusters of *n* atoms which sit on a distinct exchange site in the lattice (c.f. Inden 2001). For example, if the energetics of the A–B binary system can be described by short-range bonding in four-atom clusters, there are five possible combinations of atoms: AAAA, AAAB, AABB, ABBB, and BBBB. Ferreira et al. (1988) argued that to a first approximation, mixed clusters (AAAB, AABB, and ABBB in our simple example) are completely relaxed at standard state conditions. The relaxed elastic energy of the solid solution at the composition corresponding to cluster *c* (given by endmember proportions $p_{ci}$) can be obtained by solving Eq. 16 for $V_c$ at standard state conditions ($[P_0, T_0]$), and then substituting into the following expression (modified from Eq. 14):

23 $$\begin{aligned} \mathcal {F}_{\text {elastic,}c} = \sum _i p_{ci} \left( \mathcal {F}_i(V_c, T) - \mathcal {F}_i(V_{0i}, T) \right) . \end{aligned}$$

The total excess energy $\mathcal {F}_{\text {excess}}$ of a solid solution of composition *X* can be then determined by multiplying the relaxed elastic energy of each cluster by the probabilities of finding each cluster type in the solution $\mathrm{Pr}(c, X)$ and adding a term accounting for the configuration entropy:

24 $$\begin{aligned} \mathcal {F}_{\text {excess}}(V, T, X)= \sum _{c=1}^{m} \mathrm{Pr}(c, X) \mathcal {F}_{\text {excess},c} - T S_{\text {conf}}(V, T, X) \end{aligned}$$

25 $$\begin{aligned} \mathcal {F}_{\text {excess},c}= -\mathcal {F}_{\text {elastic,}c} + \mathcal {F}_{\text {nonelastic,}c}, \end{aligned}$$

where $\mathcal {F}_{\text {nonelastic,}c}$ is a term accounting for any other chemical effects affecting the free energy of each cluster. The probabilities are found by minimising the Helmholtz free energy subject to the bulk composition constraints. Descriptions of how to calculate $S_{\text {conf}}$ from the proportions of clusters using the cluster variational method (CVM) and the more easily extensible cluster site approximation (CSA) can be found in Inden (2001).

In a solid solution with a completely disordered site with elemental proportions $[P_A, P_B, \ldots , P_L]$, the probability $\mathrm{Pr}(c)$ of a cluster with composition $[x_A, x_B,\ldots , x_L]$ (where $x_i$ is the number of atoms of type *i*) is given by the multinomial distribution:

26 $$\begin{aligned} \mathrm{Pr}(c) = \frac{n!}{x_A!x_B!\cdots x_L!}P_A^{x_A}P_B^{x_B}\cdots P_L^{x_L}. \end{aligned}$$

In the special case of a disordered binary solution with quadratic elastic energies of mixing, and where non-elastic excesses are negligible, the excess non-configurational energy is a fixed proportion of the total unrelaxed elastic energy (Appendix 1 in Ganguly et al. 1993):

27 $$\begin{aligned} \mathcal {F}_{\text {excess}}(V, T, X) = -\left( \frac{n - 1}{n}\right) \mathcal {F}_{\text {elastic}}(X) - T S_{\text {conf}}(X). \end{aligned}$$

#### Relationship with macroscopic models of order–disorder

The insights obtained from the microscopic treatment in “Short-range clustering” can be related to generalised Bragg–Williams models of order–disorder (Ghiorso 1990; Ghiorso et al. 1995; Holland and Powell 1996; Ghiorso and Evans 2002; Holland and Powell 2006). These macroscopic models consider order–disorder processes via mixing of endmember phases and intermediate ordered compounds. In the compact symmetric formalism of Holland and Powell (1996), the state of order in a binary $A_n$–$B_n$ system with an ordered phase *O* ($O=A_rB_{n-r}$) is obtained by solving the equilibrium relationship:

28 $$\begin{aligned} 0 = a + bQ + cX + RT \ln K_\mathrm{D}, \end{aligned}$$

where the order parameter *Q* is equal to the proportion of the ordered phase ($p_O = 1 - p_A - p_B$) and

29 $$\begin{aligned} a =&\varDelta \mathcal {G}_R + n W_{\mathcal {G}}^{AO} - (n-r)W_{\mathcal {G}}^{AB} \end{aligned}$$

30 $$\begin{aligned} b =&\frac{2}{n} \Big (-r^2 W_{\mathcal {G}}^{AO} - (n-r)^2 W_{\mathcal {G}}^{BO} \end{aligned}$$

31 $$\begin{aligned}&+ r(n-r) \left( W_{\mathcal {G}}^{AB} - W_{\mathcal {G}}^{AO} - W_{\mathcal {G}}^{BO} \right) \Big ) \end{aligned}$$

32 $$\begin{aligned} c =&r \left( W_{\mathcal {G}}^{BO} - W_{\mathcal {G}}^{AO} - W_{\mathcal {G}}^{AB} \right) \end{aligned}$$

33 $$\begin{aligned}&+ (n-r) \left( W_{\mathcal {G}}^{BO} - W_{\mathcal {G}}^{AO} + W_{\mathcal {G}}^{AB} \right) \end{aligned}$$

34 $$\begin{aligned} K_\mathrm{D} =&\frac{(1 - p_A)(1 - p_B)}{p_A p_B}. \end{aligned}$$

The reaction energy $\varDelta \mathcal {G}_R$ corresponds to that required to create *n* moles of the ordered phase from the endmembers ($(rA_n + (n-r)B_n) \rightarrow nO$). The interaction parameters $W_{\mathcal {G}}^{ij}$ are derived from laboratory experiments.

As noted in the previous section, within solid solutions, the Helmholtz free energy is a more natural potential to use than the Gibbs free energy. The above expressions remain valid if $\mathcal {G}$ is exchanged for $\mathcal {F}$, because $\left( \frac{\partial \mathbf {\mathcal {G}}}{\partial \mathbf {x}} \right) _{P} = \left( \frac{\partial \mathbf {\mathcal {F}}}{\partial \mathbf {x}} \right) _{V}$ (see Appendix C). The rest of this section shows how the elastic model can be used to provide parameter estimates for the Bragg–Williams model evaluated at constant volume and temperature.

The mixed-atom clusters in “Short-range clustering” can be considered the microscopic counterparts to a unique set of ordered compounds. For example, in the four-site cluster considered in the previous section, a solid solution of composition $\hbox {AB}_3$ at 0 K will be composed entirely of ABBB clusters (as long as the ABBB cluster is more stable than any linear combination of the other clusters). The Helmholtz energy is linear between adjacent stable compounds at 0 K, because mixing between these compounds involves intercluster bonding not considered in the model in “Short-range clustering”. These observations provide a set of heuristics for the macroscopic model:

35 $$\begin{aligned} \varDelta \mathcal {F}_R= n \mathcal {F}_{\text {excess,}O} \end{aligned}$$

36 $$\begin{aligned} W_{\mathcal {F}}^{AO}= W_{\mathcal {F}}^{BO} = 0, \end{aligned}$$

where $\mathcal {F}_{\text {excess,}O}$ is the excess Helmholtz free energy of the ordered compound (Eq. 25). The factor *n* in the first expression arises from the *n* moles of the ordered phase created in the reaction $rA + (n-r)B \rightarrow nO$. If $n=2$ and the excess energy is quadratic with composition (a reasonable assumption except in cases of extreme non-ideality), then complete disordering at high temperature requires that:

37 $$\begin{aligned} W_{\mathcal {F}}^{AB} = 4(\mathcal {F}_{\text {excess,}O})/2) = 2 \mathcal {F}_{\text {excess,}O}. \end{aligned}$$

This expression uses Eq. 26 to deduce that the proportion of the mixed cluster at the midpoint of the binary is 0.5 when the solution is completely disordered. If $n>2$, stabilising a single ordered phase (*O* = $A_rB_{n-r}$) at 0 K requires non-zero non-elastic energies ($\mathcal {F}_{\text {nonelastic,}c}$) to destabilise the other potential ordered phases. The excess energy associated with each cluster can, therefore, not be quadratic as a function of composition and there is no obvious way to estimate an appropriate value of $W_{\mathcal {F}}^{AB}$. The only requirement is that the ordered phase is more stable than the antiordered phase, which is only true if

38 $$\begin{aligned} W_{\mathcal {F}}^{AB}> \frac{n^2}{2r(n-r)} \mathcal {F}_{\text {excess,}O}. \end{aligned}$$

### Calculating excesses and activities at fixed pressure and temperature

A common use for thermodynamic solution models is in phase equilibrium calculations. These calculations solve (either directly or indirectly) the equilibrium relations for the independent set of endmember reactions:

39 $$\begin{aligned} 0 = \sum n_i \mu _i, \end{aligned}$$

where $n_i$ is the number of moles of endmember *i* in the reaction, and $\mu _i$ is the chemical potential (or partial Gibbs free energy) of that endmember. For a solution with bulk composition $\mathbf {x}$, and free energy $\mathcal {G}$:

40 $$\begin{aligned} \mu _i(P, T)= \mathcal {G}(P, T, X) + \left( \frac{\partial \mathbf {\mathcal {G}}(P, T, X)}{\partial \mathbf {x}} \right) _{P} \cdot (\mathbf {x}_i - \mathbf {x}). \end{aligned}$$

The elastic model is described in terms of the Helmholtz energy at constant volume and temperature, and so calculation of the chemical potentials of the endmembers at constant pressure requires the following steps:

1. Find the equilibrium volume for a solid solution of a given composition by solving Eq. 16.
2. Find the Gibbs free energy $\mathcal {G}$ by inserting the equilibrium volume into Eq. 14 and then using the Legendre transformation $\mathcal {G} = \mathcal {F} + PV$.
3. Calculate the compositional partial derivative of the Gibbs free energy $\left( \frac{\partial \mathbf {\mathcal {G}}}{\partial \mathbf {x}} \right) _{P}$. This can be computed by differentiation of Eq. 14 with respect to composition, because $\left( \frac{\partial \mathbf {\mathcal {G}}}{\partial \mathbf {x}} \right) _{P} = \left( \frac{\partial \mathbf {\mathcal {F}}}{\partial \mathbf {x}} \right) _{V}$ (see Appendix C).
4. Find $$\mu$$.

## Applications

### Room-condition excess enthalpies in the alkali halides

The endmembers of the B1-structured alkali halides (K,Na,Rb)(Cl,Br,I) have volumes from $27.0\ \hbox {cm}^{3}/\hbox {mol}$ (NaCl) to $59.6\ \hbox {cm}^{3}/\hbox {mol}$ (RbI) at standard state. At room temperature, their bulk moduli lie between 10 and 25 GPa, with the larger bulk moduli typically corresponding to the endmembers with the smaller volumes (Dewaele et al. 2012; Dorogokupets and Dewaele 2007; Chang and Barsch 1971; Sato-Sorensen 1983; Sceats et al. 2006). The large range of endmember volumes make these simple compounds a useful system for testing theoretical models of solid solution excesses.

**Fig. 1:**
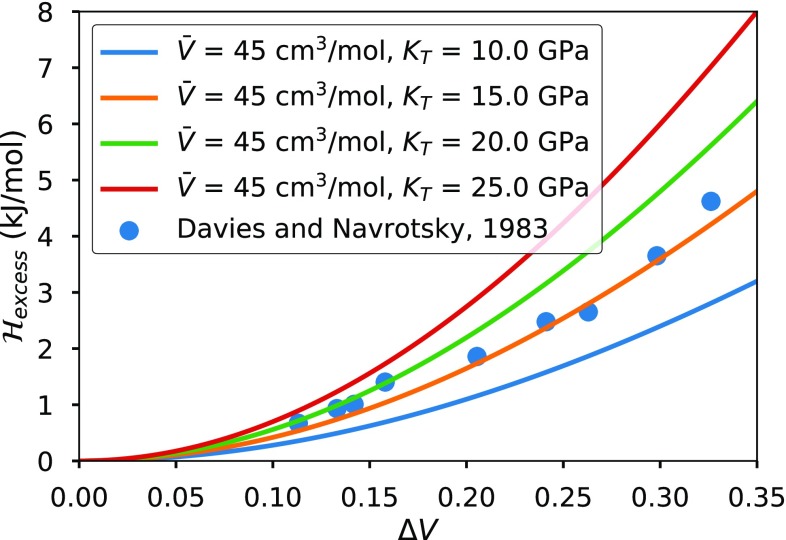
Excess enthalpies of $A_{50}B_{50}$ alkali halide solutions as a function of the difference in volumes between endmembers ($\varDelta V = 2|(V_1 - V_2)/(V_1 + V_2)|$). Solid lines correspond to the predictions of the elastic model using the Rydberg/Vinet equation of state (Rydberg 1932; Vinet et al. 1986) with $K'=5.5$. A cluster size of 2 is used, which is equivalent to assuming complete relaxation of every bond pair between dissimilar cations or anions (e.g., K–Cl–Na)

Interaction enthalpies and volume differences ($\varDelta V$) of the alkali halides have been compiled by Davies and Navrotsky (1983). These data are plotted in Fig. 1, along with the excess enthalpies predicted by the elastic model. The observed interaction energies compiled by Davies and Navrotsky (1983) are in good agreement with the model extrapolations given that all but two of the nine binaries (KI–RbI and KCl–NaCl) have mean bulk moduli between 15 and 20 GPa. The relationship between $\varDelta V$ and excess enthalpy $\mathcal {H}_{\text {excess}}$ is almost quadratic. Elastic model predictions (Urusov 2000; Geiger 2001, this study) are nearly quadratic, because the volume change of the endmembers can be well approximated by compression and expansion of bonds with harmonic potentials.

### High-pressure excesses in jadeite–aegirine pyroxenes

**Fig. 2:**
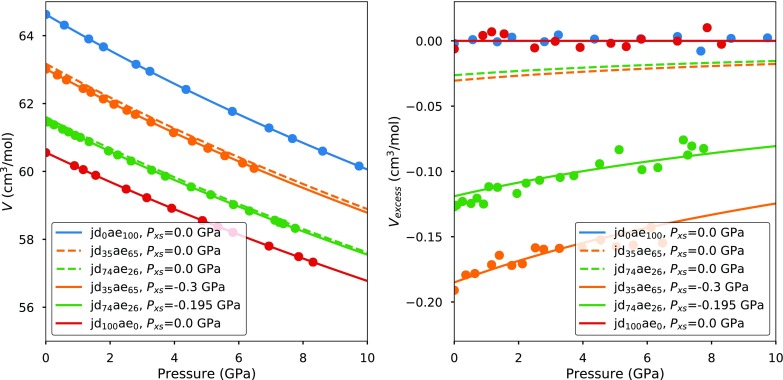
Pressure–volume data in the binary system jadeite–aegirine (Nestola et al. 2006). Dashed lines correspond to volumes predicted by the zero-parameter elastic model based on the endmember properties of jadeite and aegirine. Solid lines correspond to the models after applying a small excess pressure to each composition (Table 1). Note that this single parameter provides an excellent fit to the equations of state, including the decay of excess volumes with pressure

Elastic solid solution models can be evaluated at any *P*–*T* conditions of interest, as long as endmember equations of state are available. Figure 2 shows the isotropic elastic model predictions for jadeite–aegirine ($\hbox {Na}(\hbox {Al},\hbox {Fe})\hbox {Si}_2\hbox {O}_6$) room-temperature equations of state, based on the endmember equations of state provided in Table 1.

**Table 1:** Best-fit Rydberg/Vinet equation of state parameters for jadeite–aegirine pyroxenes from the room-temperature data of Nestola et al. (2006)

Composition	$\mathcal {V}_0$ ($\hbox {cm}^3/\hbox {mol}$)	$K_0$ (GPa)	$K'_0$	$P_{\text {excess}}$ (GPa)
$\hbox {jd}_{0}\hbox {ae}_{100}$ (fit)	$64.626 \pm 0.003$	$116.0 \pm 0.4$	$4.5 \pm 0.1$	
$\hbox {jd}_{35}\hbox {ae}_{65}$ (fit)	$63.017 \pm 0.003$	$124.3 \pm 0.2$	$3.9 \pm 0.2$	
$\hbox {jd}_{35}\hbox {ae}_{65}$ (fit)	$63.021 \pm 0.002$	$122.9 \pm 0.2$	4.5 [fixed]	
$\hbox {jd}_{35}\hbox {ae}_{65}$ (model)	63.019	123.2	4.48	$-0.300$
$\hbox {jd}_{74}\hbox {ae}_{26}$ (fit)	$61.492 \pm 0.002$	$130.4 \pm 0.5$	$4.5 \pm 0.2$	
$\hbox {jd}_{74}\hbox {ae}_{26}$ (model)	61.499	129.9	4.49	$-0.195$
$\hbox {jd}_{100}\hbox {ae}_{0}$ (fit)	$60.561 \pm 0.003$	$133.9 \pm 0.7$	$4.6 \pm 0.2$	
$\hbox {jd}_{100}\hbox {ae}_{0}$ (fit)	$60.559 \pm 0.002$	$134.5 \pm 0.2$	4.5 [fixed]	

Fitting is conducted using the automated routines in the software package *burnman* (Cottaar et al. 2014)

The model with constant $\mathcal {F}_{\text {excess}}$ (dashed lines in Fig. 2) underestimates the magnitude of the negative excess volumes at zero pressure. It is, therefore, necessary to apply a negative excess pressure to each solution (constant $\mathrm{d}\mathcal{F}_{\text {excess}}/\mathrm{d}V$ in Eq. 16). The resulting equations of state provide an excellent fit to the observed excess volumes (solid lines in Fig. 2). The evolution of the equation of state across the binary can be approximated by the following subregular model:

41 $$\begin{aligned} P_{\text {excess}}(V, T) = p_{\text {jd}}\,p_{\text {aeg}}^2 \, W_{\text {jd,aeg}}^P + p_{\text {aeg}}\,p_{\text {jd}}^2 \,W_{\text {aeg,jd}}^P, \end{aligned}$$

where $W_{\text {jd,aeg}}^P = -1.6$ GPa and $W_{\text {jd,aeg}}^P = -0.8$ GPa. This example demonstrates that a good approximation to solid solution equations of state can be obtained with the isotropic elastic model using fewer parameters than the conventional Gibbs formulations, which would require a pressure-dependent excess volume term.

The need for an empirical excess pressure term to fit jadeite–aegirine data to the isotropic elastic model is not surprising, as the endmembers are monoclinic and have structural flexibility which is not considered in the simple model. The excess pressure term probably reflects the fact that negative excess volumes across the solution (relative to the elastic model) should inhibit changes in tilting of adjacent $\hbox {SiO}_4$-tetrahedra (Boffa Ballaran et al. 1998), decreasing the compressibility. This hypothesis is supported by the data of Nestola et al. (2006), which shows that compressional anisotropy rapidly decreases with increasing jadeite content (their Fig. 1e).

### High *P*–*T* excesses in garnet

It has been suggested that elastic energies dominate non-ideality in garnet solid solutions (Ganguly et al. 1996; Boffa Ballaran et al. 1999; Bosenick et al. 2001). Indeed, the garnet structure cannot accommodate compression via pure rotations of structural units (Hammonds et al. 1998), so the formation of solutions between dissimilar endmembers must involve a significant amount of bond strain (Boffa Ballaran et al. 1999). One of the most-studied garnet solutions is the pyrope-grossular binary ($(\hbox {Mg},\hbox {Ca})_3\hbox {Al}_2\hbox {SiO}_{12}$). In comparison with the nearly ideal $\hbox {Mg}^{2+}$–$\hbox {Fe}^{2+}$ exchange in the pyrope–almandine system (Ganguly et al. 1996; White et al. 2014), the exchange of Mg$^{2+}$ for the much larger Ca$^{2+}$ cation (ionic radii of 0.89 and 1.12 Å  respectively; Shannon 1976) on the dodecahedral (X) site leads to large excess enthalpies and even exsolution at low temperatures (Cressey 1978; Wang et al. 2000). However, published studies do not agree on the compositional dependence of the thermodynamic excesses across the binary (Table 2).

**Table 2:** Maximum model excesses (and the composition of the maximum excess) in the pyrope–grossular system at 1 bar and 300 K according to several published studies

$\mathcal {H}_{\text {excess}}$ (kJ/mol)	$V_{\text {excess}}$ ($\hbox {cm}^3/\hbox {mol}$)	$S_{\text {excess}}$ (J/K/mol)	References
12.0 ($\hbox {py}_{60}$)	0.25 ($\hbox {py}_{50}$)	–	Berman (1990)
08.5 ($\hbox {py}_{40}$)	0.30 ($\hbox {py}_{35}$)	–	Ganguly et al. (1993)
06.5 ($\hbox {py}_{60}$)	0.35 ($\hbox {py}_{60}$)	0 (assumed)	Green et al. (2012)
–	1.00 ($\hbox {py}_{50}$)	–	Du et al. (2015)
–	–	$4.7 \pm 0.5$ ($\hbox {py}_{50}$)	Haselton and Westrum (1980)
–	–	$2.9 \pm 0.5$ ($\hbox {py}_{50}$)	Dachs and Geiger (2006)

**Fig. 3:**
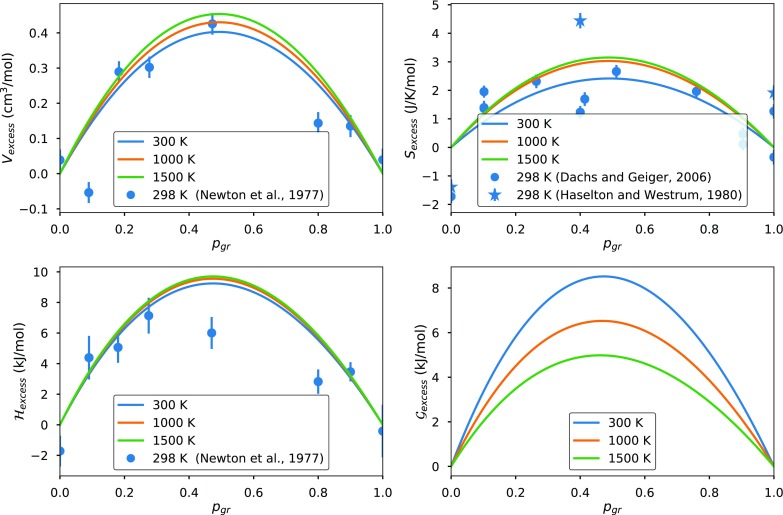
Non-configurational pyrope–grossular excesses for a disordered, purely elastic solution model (with energy corrected for relaxed 3-atom clusters, as described in “The chemical contribution to the excess Helmholtz energy”) calculated at 1 bar. Plotted uncertainties in the experimental data correspond to the measured values, rather than the excesses. The conversion from the measured values to excess properties has been estimated by fitting a quadratic function to all the datapoints and removing the linear component of that function

Here, I construct a disordered elastic model from the pyrope and grossular endmember equations of state proposed by Stixrude and Lithgow-Bertelloni (2011). The excess Helmholtz free energy ($\mathcal {F}_{\text {excess}}$; Eq. 14) is calculated assuming that the bonds within Mg–Mg–Ca and Mg–Ca–Ca clusters are fully relaxed at the standard state equilibrium volume (i.e., $n = 3$ in “The chemical contribution to the excess Helmholtz energy”) as proposed by Ganguly et al. (1993). The 1 bar predictions computed from this model are in good agreement with the experimental excesses reported in the literature (Newton et al. 1977; Haselton and Westrum 1980; Ganguly et al. 1993; Dachs and Geiger 2006), as shown in Fig. 3. Particular noteworthy are the excess entropy predictions, which agree well with recent calorimetric data (Dachs and Geiger 2006). The excess entropies in the elastic model are a consequence of the volume dependence of the endmember densities of state (Stixrude and Lithgow-Bertelloni 2011), and, therefore, arguably represent the most conservative model.

**Fig. 4:**
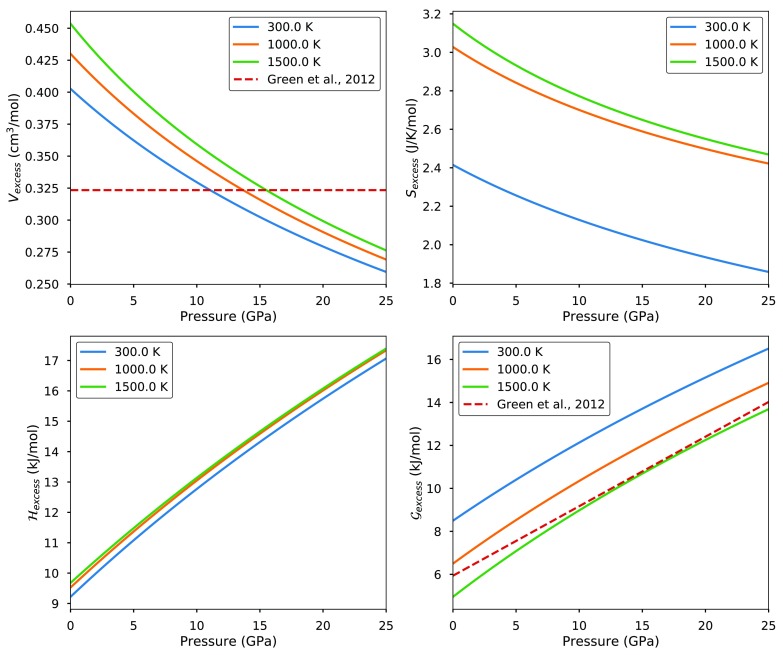
Non-configurational $\hbox {py}_{50}\hbox {gr}_{50}$ excesses for a disordered, purely elastic solution model (with energy corrected for relaxed 3-atom clusters, as described in “The chemical contribution to the excess Helmholtz energy”) as a function of pressure

Figure 4 shows the elastic model predictions for the $\hbox {py}_{50}\hbox {gr}_{50}$ composition at high pressures and temperatures. At 1000–1500 K, the excess Gibbs free energy predicted by the model is in remarkably good agreement with the empirical fit proposed by Green et al. (2012) on the basis of phase equilibria. The non-configurational excess entropy results in systematic deviations from Green et al. (2012) at lower and higher temperatures.

Although the model fits the data well, it differs from all of the published models compiled in Table 2. The key difference is that the elastic model excesses are almost perfectly quadratic as a function of composition. Given the scatter in the experimental data, and the disagreement between existing models, this is probably reasonable. Any strong asymmetry in excess properties may be the result of ordering (e.g., Newton and Wood 1980), or microstrain (e.g., Du et al. 2015). Whatever the cause, the elastic model may be a more robust predictor of excess properties at mantle conditions (where deviatoric stresses are low and elements are largely disordered on the dodecahedral site) than empirical models calibrated at low temperatures.

**Fig. 5:**
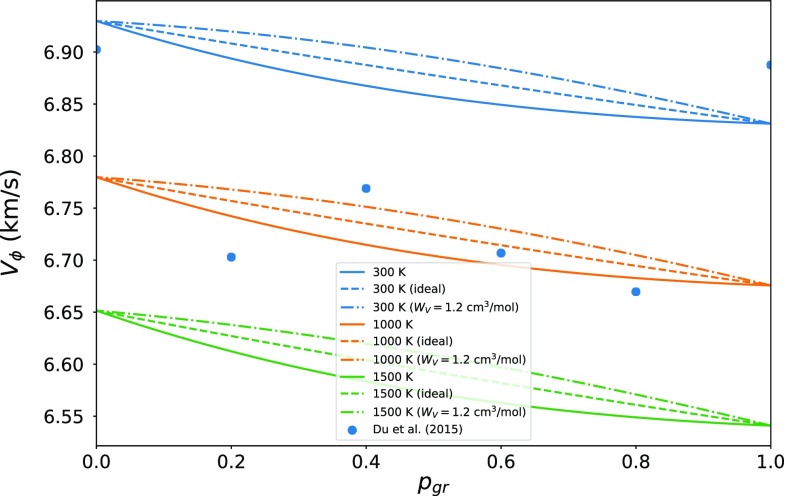
Model bulk sound velocities along the pyrope–grossular join at 300, 1000, and 1500 K. The models plotted are an ideal solution (dashed lines), a model with a constant volume excess (dot-dashed lines), and the elastic model described in the text (solid lines). Data points are calculated from the experimentally derived 300 K equations of state of Du et al. (2015), with isentropic bulk moduli calculated using the thermodynamic identity $K_\mathrm{S} = K_T(1 + \alpha \gamma T)$ and the thermal expansion and Grüneisen parameter taken from an ideal pyrope–grossular solution (the choice of model is unimportant at 300 K)

Finally, the elastic model can be used to predict seismic velocities across the pyrope–grossular binary (Fig. 5). The positive excess volume and negative excess bulk modulus (Fig. 4) induce a small negative deviation from ideal bulk sound speeds, in contrast to constant excess volume models, which predict a positive deviation. The elastic model predictions are in disagreement with Du et al. (2015), whose results imply a large decrease in bulk sound speed across the solid solution. To fit the volume excesses of Du et al. (2015) within the elastic model framework, an excess pressure of $\sim 1$ GPa is required in the center of the binary, while to fit the bulk sound speed, the excess pressure must be on the order of 2.5 GPa. Simultaneously fitting the bulk modulus and volume would require large values of $\partial ^2 \mathcal {F}_{\text {excess}}/\partial V^2$. Given the success of the elastic model in predicting the room pressure volumetric and thermal properties (Fig. 3), it seems unlikely that such large values are reasonable.

### The bridgmanite solid solution

Bridgmanite is a magnesium silicate mineral with the $\hbox {CaTiO}_3$-perovskite structure (Pbnm space group; Tschauner et al. 2014). It is the dominant mineral in the Earth’s lower mantle, playing a key role in determining the seismic velocities and density structure of the deep mantle from the 660 km discontinuity until its breakdown to the $\hbox {CaIrO}_3$-type post-perovskite structure a few hundred kilometers above the core-mantle boundary (Murakami et al. 2004). The compositions of mantle bridgmanites can broadly be described by the general formula $\hbox {ABO}_3$, where $\hbox {A} = (\hbox {Mg}^{2+}, \hbox {Fe}^{2+}, \hbox {Fe}^{3+}, \hbox {Al}^{3+}$), and $\hbox {B} = (\hbox {Al}^{3+}, \hbox {Si}^{4+})$ (Frost and Myhill 2016). Minor components include CaSiO$_3$ and $\hbox {Na}_{0.5}\hbox {Al}_{0.5}\hbox {SiO}_3$ (Litasov et al. 2004; Holland et al. 2013). The four most important endmembers of bridgmanite are, therefore, $\hbox {MgSiO}_3$, $\hbox {FeSiO}_3$, $\hbox {AlAlO}_3$, and $\hbox {FeAlO}_3$, although $\hbox {MgSiO}_3$ is the only endmember which is stable in the bridgmanite structure (Wicks and Duffy 2016).

Within this simplified bridgmanite system, only the $\hbox {MgSiO}_3$–$\hbox {FeSiO}_3$ and $\hbox {AlAlO}_3$–$\hbox {FeAlO}_3$ binaries involve exchange on a single site. The other four involve coupled exchange to maintain charge balance. This is an important point, as the assumptions of bond relaxation in “The chemical contribution to the excess Helmholtz energy” rely on exchange on a single site in the lattice. Coupled exchange may be associated with non-negligible energy costs. For example, in the $\hbox {MgSiO}_3$–$\hbox {FeAlO}_3$ binary eightfold coordinated $\hbox {Mg}^{2+}$ will have a larger ionic radius than $\hbox {Fe}^{3+}$, while sixfold coordinated $\hbox {Si}^{4+}$ will have a smaller ionic radius than $\hbox {Al}^{3+}$ (Shannon 1976). Complete relaxation is, therefore, unlikely for either $\hbox {Fe}^{3+}$–O–$\hbox {Si}^{4+}$ (smaller cations) or $\hbox {Mg}^{2+}$–O–$\hbox {Al}^{3+}$ (larger cations) bond structures. Nevertheless, the two bond structures are likely to have competing effects on the equilibrium volume. For this reason, I focus on the elastic model predictions for the excess volumes and bulk moduli within the solid solution, given endmember properties derived from the literature (Table 3). $\hbox {MgSiO}_3$ properties are chosen to fit experimental data (Fiquet et al. 2000; Murakami et al. 2007). The $\hbox {FeSiO}_3$ and $\hbox {AlAlO}_3$ properties are extrapolated from $\hbox {MgSiO}_3$ using the trend in volume and bulk modulus reported by Caracas and Cohen (2005). The $\hbox {FeAlO}_3$ properties represent a small thermal adjustment from Caracas (2010).

**Table 3:** Properties of bridgmanite at 300 K (third-order Birch–Murnaghan equation of state)

Composition	$V_0$ ($\hbox {cm}^3/\hbox {mol}$)	$K_0$ (GPa)	$K'_0$
$\hbox {MgSiO}_3$ (this study)	24.45	$253 \pm 2$	$3.90 \pm 0.10$
$\hbox {FeSiO}_3$ (adjusted from CC2005)	24.88	251	3.90
$\hbox {AlAlO}_3$ (adjusted from CC2005)	25.91	223	4.03
$\hbox {FeAlO}_3$ (adjusted from C2010)	27.68	207	3.73
$(\hbox {MgSi})_{0.87}(\hbox {FeSi})_{0.13}\hbox {O}_3$ (W2015)	$24.50 \pm 0.05$ (24.50)	$253 \pm 4$ (253)	3.9 [fixed] (3.90)
$(\hbox {MgSi})_{0.75}(\hbox {AlAl})_{0.25}\hbox {O}_3$ (W2004)	$24.81 \pm 0.01$ (24.80)	$256 \pm 2$ (245)	3.9 [fixed] (3.90)
$(\hbox {MgSi})_{0.9}(\hbox {FeAl})_{0.1}\hbox {O}_3$ (K2017)	$24.74 \pm 0.02$ (24.76)	$247 \pm 1$ (247)	3.7 (3.88)

The values $\hbox {MgSiO}_3$ provide a good fit to Fiquet et al. (2000) and Murakami et al. (2007). The $(\hbox {MgSi})_{0.87}(\hbox {FeSi})_{0.13}\hbox {O}_3$, $(\hbox {MgSi})_{0.75}(\hbox {AlAl})_{0.25}\hbox {O}_3$, and $(\hbox {MgSi})_{0.9}(\hbox {FeAl})_{0.1}\hbox {O}_3$ equations of state are fit to data from Wolf et al. (2015), Walter et al. (2004), and Kurnosov et al. (2017), respectively (see text)

**Fig. 6:**
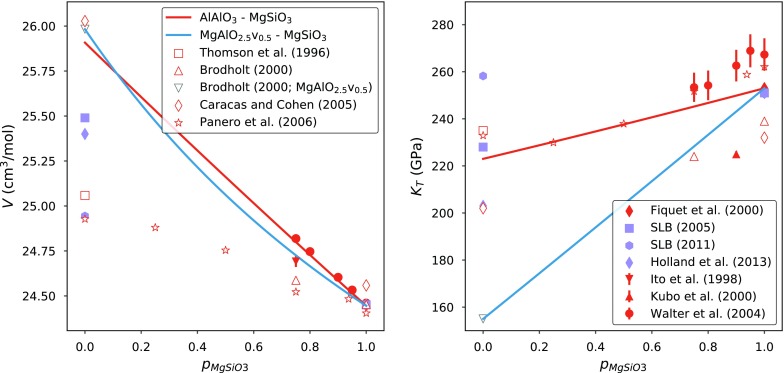
Elastic properties of the stoichiometric $\hbox {MgSiO}_3$–$\hbox {AlAlO}_3$ and defect-structured $\hbox {MgSiO}_3$–$\hbox {MgAlO}_{2.5}\hbox {v}_{0.5}$ binaries. Ab initio data are shown as open symbols (Thomson et al. 1996; Brodholt 2000; Caracas and Cohen 2005; Panero et al. 2006). Experimental data are shown as red closed symbols (Fiquet et al. 2000; Ito et al. 1998; Kubo et al. 2000; Walter et al. 2004). Data set values are shown as purple closed symbols (Stixrude and Lithgow-Bertelloni 2005, 2011; Holland et al. 2013). Red and blue lines correspond, respectively, to elastic models for the $\hbox {MgSiO}_3$–$\hbox {AlAlO}_3$ and $\hbox {MgSiO}_3$–$\hbox {MgAlO}_{2.5}\hbox {v}_{0.5}$ binaries

The modelled elastic properties for solutions along each binary are mostly within error of experimentally derived values (also listed in Table 3). The exceptions are the bulk modulus of $\hbox {AlAlO}_3$-bearing bridgmanite and the $K'$ of $\hbox {FeAlO}_3$ bridgmanite. The elastic behaviour of the $\hbox {MgSiO}_3$–$\hbox {AlAlO}_3$ binary is extremely difficult to characterise experimentally (Fig. 6). Samples with similar bulk compositions produce estimates of bulk moduli spanning about 20 GPa (Daniel et al. 2001, 2004; Walter et al. 2004; Yagi et al. 2004; Andrault et al. 2007). This may be the result of high compressibility of defect-structured bridgmanite or sensitivity to deviatoric stress (Andrault et al. 2007). The elastic model with (adjusted) ab initio endmember properties may, therefore, present a more appropriate model for mixing than the experimental data. Brillouin measurements of 5 mol% $\hbox {AlAlO}_3$-bearing bridgmanite (Jackson et al. 2004) are in good agreement with the elastic model.

**Fig. 7:**
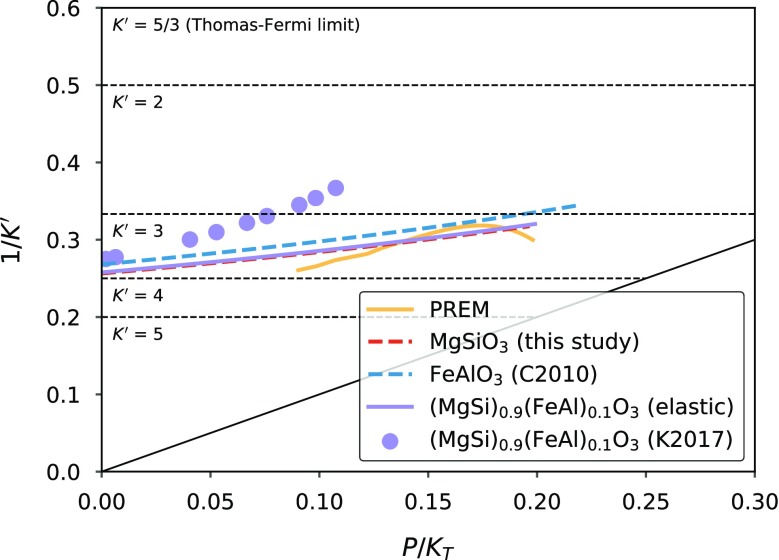
Pressure dependence of $K'$ for $\hbox {MgSiO}_3$ bridgmanite (this study), $\hbox {FeAlO}_3$ bridgmanite (minor thermal adjustment to simulation “AFM12” from Caracas 2010) using third-order Birch-Murnaghan equations of state up to 150 GPa, and the elastic model predictions for the $(\hbox {MgSi})_{0.9}(\hbox {FeAl})_{0.1}\hbox {O}_3$ solid solution. The elastic model predictions clearly diverge from experimental Brillouin measurements up to 40 GPa (Kurnosov et al. 2017). Similar experiments on $\hbox {MgSiO}_3$ (Murakami et al. 2007) are incompatible with a similar decrease in $K'$, and as a result, the experimental data on $\hbox {MgSiO}_3$ and $(\hbox {MgSi})_{0.9}(\hbox {FeAl})_{0.1}\hbox {O}_3$ cannot be simultaneously fit with any physically plausible elastic model (see text). Also shown are values of $K'$ for the Earth inferred from the Preliminary Reference Earth Model (Dziewonski and Anderson 1981). The 1:1 line represents the loci of possible infinite-pressure values, and the upper edge of the plot represents a theoretical limit on $K'$ (Stacey and Davis 2004)

The experimental values for $K'$ of the $\hbox {MgSiO}_3$–$\hbox {FeAlO}_3$ solution (Kurnosov et al. 2017) have major implications for both the seismic velocities in the lower mantle, and our ability to thermodynamically model the bridgmanite solid solution. Figure 7 shows the experimental data in $K'$-space (Stacey and Davis 2004), where it becomes clear that $K'$ increasingly diverges from the $\hbox {MgSiO}_3$ trend (Fiquet et al. 2000; Murakami et al. 2007), and from the $K'$ of the lower mantle. Extrapolation from the experimental $\hbox {MgSiO}_3$ and $(\hbox {MgSi})_{0.9}(\hbox {FeAl})_{0.1}\hbox {O}_3$ data requires increasing non-ideality with pressure to avoid negative values of $K'$ for the $\hbox {FeAlO}_3$ endmember (see Eq. 21). In the context of the elastic model, this non-ideality would have to include a large $\partial ^2 \mathcal {F}/\partial V^2$ component that changes sign at small volumes to ensure plausible values of $K'$ ($>5/3$; Stacey and Davis 2004) across the binary. The alternative is that the high-pressure experimental data may have been affected by deviatoric stresses, as previously invoked to explain data on the $\hbox {MgSiO}_3$–$\hbox {AlAlO}_3$ binary (Andrault et al. 2007). Given the excellent agreement between the ab initio and elastic model predictions, and the agreement with the low-pressure XRD and Brillouin data, the second possibility seems more likely. The implication is that seismic studies of the Earth’s lower mantle need not consider excess volumes or large $K'$ variations in bridgmanite solid solutions.

## Discussion

Elastic models of solid solutions are based on the idea that excess nonconfigurational energies of formation are dominated by the endmember lattice distortions required to make the solution, and partial relaxation due to changes in bonding (c.f. Geiger 2001). As strain is the independent variable, mixing in the elastic model is best understood by considering the Helmholtz free energy, rather than the Gibbs free energy. This concept is certainly not new, despite being rarely used in the geological literature. Indeed, the origins of common order–disorder models lie in the work of Landau (1937), who used Helmholtz free energies when considering solid–solid transformations. The prevalence of solution models based on the Gibbs free energy presumably derives from the “ideal” mixing model (itself based on mixing in gases) and its ease of use when considering systems where pressure and temperature are the independent variables. Indeed, Dove (1997) used the Gibbs free energy only as a connection to experiment, noting that the Helmholtz energy was strictly the more correct potential to use. Given that some mineral databases now use equations of state based on the Helmholtz energy (Stixrude and Lithgow-Bertelloni 2005, 2011), it may, in some cases, be convenient to use elastic models directly. Even if Gibbs formulations are still used, the elastic model can provide heuristic values for poorly constrained model parameters. Non-elastic contributions to interaction parameters could be estimated via comparison with other phases, as proposed for models based on the Gibbs free energy (e.g., Powell et al. 2014).

In this study, I show that the elastic model formulation behaves well at both high temperatures and high pressures. The inclusion of short-range bonding contributions to the free energy can provide good predictions of excess energies in simple and more complex cubic systems (Sects. “Room-condition excess enthalpies in the alkali halides” and “High *P*–*T* excesses in garnet”). The agreement between the model and experimental excess entropies within the pyrope–grossular binary is particularly striking. The magnitude of the excesses suggests that the non-configurational Gibbs free energy decreases by almost 50% between room-temperature and typical mantle potential temperatures.

The elastic model also places stringent constraints on the variation of material properties within solid solutions which differ from those normally imposed in Gibbs formulations. For example, it has sometimes been assumed that the ratios $K_{T}/V$ or $V/K_{T}$ are linear functions of composition (e.g., Ferreira et al. 1988; Ganguly et al. 1993; Stixrude and Lithgow-Bertelloni 2005) in disordered solid solutions. This assumption implies that positive excess volumes are associated with positive excess bulk moduli, which is unintuitive and leads to increasing volume excesses at high pressure. In contrast, the elastic model predicts that excess volumes tend to zero at high pressure, and that excess non-configurational entropies tend to zero at low temperature.

There are limitations to the elastic model. The model requires that the elastic strain fields due to individual atomic exchanges overlap; this assumption breaks down in dilute alloys (Wood and Blundy 1997; Carpenter et al. 1999). Rigid rotations of structural units are not considered, although such effects may be accommodated by excess pressure terms (Sect. “High-pressure excesses in jadeite–aegirine pyroxenes”). Finally, the examples given in this paper use the isotropic approximation to the elastic model. Such a model may not be applicable to highly anisotropic minerals, especially if the endmembers of those minerals have very different lattice parameters. It is possible to generalise the equations in “The elastic model” to anisotropic materials and non-hydrostatic stress fields (Appendix A). In the future, it should be possible for systematic high-pressure elasticity studies (e.g., Fan et al. 2015; Huang and Chen 2014) to probe the limits of applicability of the isotropic model. It should also be possible to design ab initio simulations to test the full anisotropic elastic model. Such work would provide a better understanding of the variation of shear modulus within solid solutions, and more generally the effect of deviatoric stresses on the thermodynamics and elasticity of natural rocks (Hobbs and Ord 2016).

## Appendix A: Generalisation to anisotropic solutions and non-hydrostatic stress states

The model presented in “The elastic model” makes the approximations that the solid solutions are isotropic, and subjected to a hydrostatic stress field. The second approximation is likely to be appropriate at the high temperatures and low stresses present in the majority of the deep Earth, but the first is only appropriate for glasses, for which the elastic model is unlikely to hold.

The elastic model assumes that the energy required to deform individual endmembers to the same lattice structure contributes to the excess energy of the solution with that structure, with atomic exchange typically relaxing some of that energy. For anisotropic materials and stresses, the deformations required are also generally anisotropic. Thus, the generalisation of the isotropic elastic model requires the relationship between Helmholtz free energy and strain: (Holzapfel 2000):

42 $$\begin{aligned} \mathbb {C}_{{T}}(\mathbf {E}, T)= \frac{1}{V} \left( \frac{\partial ^2 \mathcal {F}(\mathbf {E}, T)}{\partial {\mathbf {E}} \partial {\mathbf {E}}}\right) _{T} \end{aligned}$$

43 $$\begin{aligned} \varvec{\sigma }(\mathbf {E}, T)= \frac{1}{V} \left( \frac{\partial \mathcal {F}(\mathbf {E}, T)}{\partial \mathbf {E}} \right) _{T}, \end{aligned}$$

where $\mathbb {C}_T$ is the isothermal stiffness tensor and $\mathbf {E}$ is the Green strain tensor. By analogy with Eq. 16, the stress state $\varvec{\sigma }$ experienced by the anisotropic solid solution with lattice parameters corresponding to a metric tensor $\mathbf {g}$ is equal to the molar weighted sum of the endmember stress states with the same lattice parameters:

44 $$\begin{aligned} \varvec{\sigma }(\mathbf {g}, T) = \sum _i p_i \varvec{\sigma }_i + \frac{1}{V} \left( \frac{\partial \mathcal {F}_{\text {excess}}}{\partial \mathbf {E}} \right) _{T}. \end{aligned}$$

Now, consider the change in stress accompanying a small symmetry-preserving change in the metric tensor $\delta \mathbf {g}$:

45 $$\begin{aligned} \delta \varvec{\sigma }= \varvec{\sigma }(\mathbf {g} + \delta \mathbf {g}, T) - \varvec{\sigma }(\mathbf {g}, T) \end{aligned}$$

46 $$\begin{aligned}= \mathbb {C}_T : \delta \mathbf {E} \end{aligned}$$

47 $$\begin{aligned}= \sum _i p_i \mathbb {C}_{Ti} : \delta \mathbf {E} + \frac{1}{V} \left( \frac{\partial ^2 \mathcal {F}_{\text {excess}}}{\partial {\mathbf {E}} \partial {\mathbf {E}}}\right) _T \delta \mathbf {E}. \end{aligned}$$

Therefore, in the anisotropic elastic model, the isothermal stiffness tensor is equal to the molar sum of endmember tensors:

48 $$\begin{aligned} \mathbb {C}_{T}(\mathbf {g}, T) = \sum _i p_i \mathbb {C}_{Ti} + \frac{1}{V} \left( \frac{\partial ^2 \mathcal {F}_{\text {excess}}}{\partial {\mathbf {E}} \partial {\mathbf {E}}}\right) _T. \end{aligned}$$

The formulation, therefore, preserves lattice symmetry between endmembers. In an hydrostatic environment, the thermodynamic properties of isotropic solutions satisfy the expressions in “The elastic model”. Furthermore, because isotropic isothermal and adiabatic shear modulus are equal to each other (Landau 1986):

49 $$\begin{aligned} G(\mathbf {g}, T) \sim \sum _i p_i G_{i}. \end{aligned}$$

For anisotropic materials, the expressions in “The elastic model” are not necessarily satisfied; Eq. 17 is one such example:

50 $$\begin{aligned} K_T(\mathbf {g}, T) \ne \sum _i p_\mathrm{i} K_{Ti} + V \left( \frac{\partial ^2 \mathcal {F}_{\text {excess}}}{\partial {V}^2} \right) _{T}. \end{aligned}$$

The accurate application of the generalised anisotropic elastic model requires knowledge of the effect of finite deviatoric stresses on the elastic constants. For small changes in unit cell ratios and angles, the response of the endmembers to non-hydrostatic stresses can be linearised (i.e., $\left( \partial \mathbb {C}_T/ \partial \varvec{\sigma }\right) _V = 0$). In these circumstances, solving Eq. 44 only requires *P*(*V*, *T*) and $\mathbb {C}_T(V, T)$ for each endmember.

## Appendix B: Approximating excess properties without solving for *V*

The elastic model equations in “The elastic model” can be solved for any endmember equations of state, but there may be occasions when an analytical approximation to the equilibrium volume is favoured over inverting for the exact solution. One such approximation can be obtained by applying the Murnaghan equation of state and Eq. 16 to a solid solution with endmember proportions $p_\mathrm{i}$ at a reference pressure $P_\mathrm{r}$:

51 $$\begin{aligned} P(V) - P_\mathrm{r}= \frac{K_0}{K'_0} \left( \left( \frac{V}{V_\mathrm{r}}\right) ^{-K'_0} - 1 \right) \end{aligned}$$

52 $$\begin{aligned} -P_{\text {excess}}(V)= \sum _i p_\mathrm{i} (P_\mathrm{i} - P_\mathrm{r}) \sim \sum _i p_\mathrm{i} \left( \frac{K_{\mathrm{ri}}}{K'_{\mathrm{ri}}} \left( \left( \frac{V}{V_{\mathrm{ri}}} \right) ^{-K'_{\mathrm{ri}}} - 1 \right) \right) . \end{aligned}$$

The subscripts "ri" indicate that the endmember property should be evaluated at the reference pressure. Rearranging for volume, and using the approximation that $K'=K'_\mathrm{r}$ (Eq. 21) for all phases, we have:

53 $$\begin{aligned} V(P)\sim & \left( \frac{\sum _i p_i K_{\mathrm{ri}} V_{\mathrm{ri}}^{K'_{\mathrm{r}}}}{\sum _i p_\mathrm{i} K_{\mathrm{ri}} - K'_\mathrm{r} P_{\text {excess}} }\right) ^{\frac{1}{K'_\mathrm{r}}} \end{aligned}$$

54 $$\begin{aligned} V_{\text {excess}}(P)= V(P) - \sum _i p_\mathrm{i} V_{\mathrm{ri}}. \end{aligned}$$

The endmember equations of state can then be evaluated at this volume to find an approximation to the Helmholtz free energy at $P_\mathrm{r}$ (Eq. 14).

## Appendix C: Proof that $\left( \frac{\partial \mathbf {\mathcal {G}}}{\partial \mathbf {x}} \right) _{P} = \left( \frac{\partial \mathbf {\mathcal {F}}}{\partial \mathbf {x}} \right) _{V}$

We start from the relation between the Helmholtz and Gibbs free energies:

55 $$\begin{aligned} \mathcal {G}= \mathcal {F} + PV \end{aligned}$$

56 $$\begin{aligned} d\mathcal {G}= \mathrm{d}\mathcal {F} + P\mathrm{d}V + V\mathrm{d}P \end{aligned}$$

57 $$\begin{aligned} \left( \frac{\partial \mathcal {G}}{\partial x} \right) _{P}= \left( \frac{\partial \mathcal {F}}{\partial x} \right) _{P} + P\left( \frac{\partial V}{\partial x} \right) _{P}. \end{aligned}$$

The relationship between $\left( \frac{\partial \mathcal {F}}{\partial x} \right) _{P}$ and $\left( \frac{\partial \mathcal {F}}{\partial x} \right) _{V}$ can be derived from the following total derivatives:

58 $$\begin{aligned} \mathrm{d}\mathcal {F}= \left( \frac{\partial \mathcal {F}}{\partial x} \right) _{V} \mathrm{d}x + \left( \frac{\partial \mathcal {F}}{\partial V} \right) _{x} \mathrm{d}V \end{aligned}$$

59 $$\begin{aligned} \mathrm{d}V= \left( \frac{\partial \mathcal {V}}{\partial P} \right) _{x} \mathrm{d}P + \left( \frac{\partial V}{\partial x} \right) _{P} \mathrm{d}x \end{aligned}$$

60 $$\begin{aligned} \left( \frac{\partial \mathcal {F}}{\partial x} \right) _{P}= \left( \frac{\partial \mathcal {F}}{\partial x} \right) _{V} + \left( \frac{\partial \mathcal {F}}{\partial V} \right) _{x}\left( \frac{\partial V}{\partial x} \right) _{P} \end{aligned}$$

61 $$\begin{aligned} \left( \frac{\partial \mathcal {F}}{\partial x} \right) _{P}= \left( \frac{\partial \mathcal {F}}{\partial x} \right) _{V} - P\left( \frac{\partial V}{\partial x} \right) _{P}. \end{aligned}$$

Substituting Eqs. 61 into 57 yields the desired equality:

62 $$\begin{aligned} \left( \frac{\partial \mathcal {G}}{\partial x} \right) _{P} = \left( \frac{\partial \mathcal {F}}{\partial x} \right) _{V}. \end{aligned}$$
